# Development of a 3D printed simulator for closed reduction of distal radius fractures

**DOI:** 10.1007/s40037-020-00609-w

**Published:** 2020-09-28

**Authors:** William Dixon, Nathaniel Miller, Georgia G. Toal, Stefanie S. Sebok-Syer, Michael A. Gisondi

**Affiliations:** 1 Department of Emergency Medicine, Stanford University, Stanford, CA USA; 2 Department of Emergency Medicine, Vanderbilt University, Nashville, TN USA; 3 Stanford University, Stanford, CA USA

**Keywords:** Distal radius fracture, Simulation based mastery learning, Medical education

## Abstract

**Background:**

The use of simulators in medical education is critical for developing procedural competence prior to treating patients. Current training of emergency physicians to perform distal radius fracture reduction is inconsistent and inadequate.

**Approach:**

We developed a 3D printed distal radius fracture simulation training model that is easy to assemble and relatively inexpensive. We present step-by-step instructions to reproduce the model.

**Evaluation:**

The model was found to have high fidelity for training by both instructors and participants in a simulation-based mastery learning course.

**Reflection:**

We successfully designed a low cost, easy to reproduce, high fidelity model for use in a simulation-based mastery learning course to teach distal radius fracture reduction.

**Electronic supplementary material:**

The online version of this article (10.1007/s40037-020-00609-w) contains supplementary material, which is available to authorized users.

## Background

The successful reduction and treatment of distal radius fractures in the emergency department is a core competency for emergency physicians. However, there are widely variable opportunities to master this skill during emergency medicine residency training in the United States. Subsequently, emergency physicians may lack the necessary expertise to teach the next generation of trainees to perform this procedure competently, leading to difficulties in standardizing training experience across the profession and perpetuating this lack of skill development in the specialty. The current approach to teaching fracture reduction can be irregular; typically, the procedure is taught at the bedside and can be limited by supervisor experience and comfort with the procedure [1]. Existing research shows both dissatisfaction with musculoskeletal training and deficiencies in musculoskeletal knowledge amongst some emergency physicians [2].

Simulation-based mastery learning improves training in bedside procedures and can serve as a theoretical framework to address training gaps [3–6]. Briefly, simulation-based mastery learning stresses asynchronous preparation, deliberate practice, close supervision during training, and a checklist assessment to ensure learning to a predetermined mastery standard. Simulation offers learners opportunities for deliberate practice of a procedure supervised by skilled educators, before performing these procedures in the actual clinical environment. Simulators for distal radius fractures are described in the orthopedic literature; however, reproducing these models requires the purchase of expensive, industrially produced replica bones [7–9]. The advent of at-home 3D printing provides the opportunity for a more generally reproducible model that can potentially increase access to simulation-based educational sessions on a wider scale.

Given the need for inexpensive, reproducible, and portable models for simulation of common procedures, this paper describes the production of a high-fidelity distal radius fracture model to teach proper reduction techniques in a simulation-based mastery learning course. Another advantage of our simulator is that it can be used to concurrently teach the hematoma block procedure, where a small amount of anesthetic is injected into the area around the fracture to decrease pain associated with the reduction. The focus of this paper is on the construction of the model, though brief course outcomes and trainee responses to use of the simulator are included to illustrate context.

## Approach

**Fig. 1:**
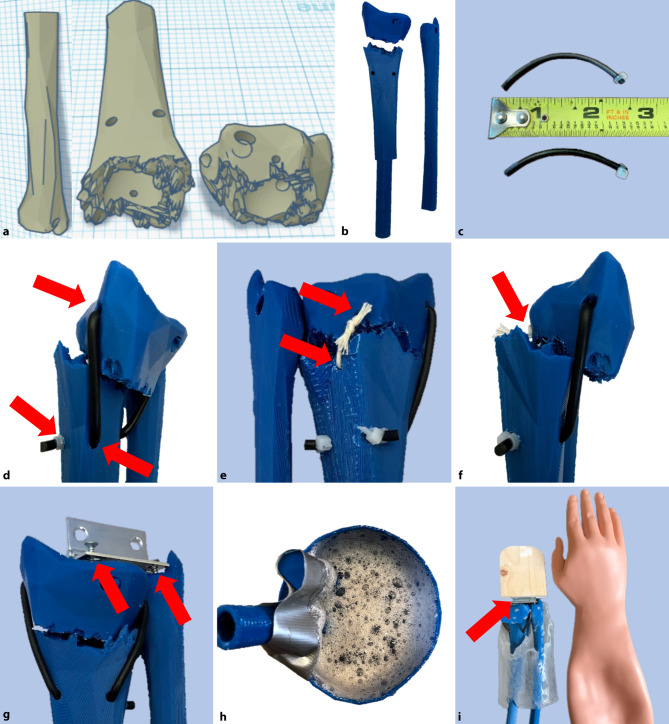
Steps in assembly of distal radius fracture model. **a** Computer Assisted Design (*CAD*) drawings of ulna and fractured radius. **b** Individual 3D printed models of ulna and fractured radius. **c** O-rings are cut into appropriate sizes and zip-ties placed at one end of each. **d** O-rings are placed through pre-printed holes in the distal fragment of radius. Second end of O‑rings is placed through pre-printed holes in the proximal radius fragment with zip-ties secured on exposed ends. **e** String is threaded through pre-printed holes on volar aspects of both fracture fragments and tied together. **f** String length is adjusted as needed until optimal displacement is achieved. **g** 1.5-inch (3.81 cm) hinges are screwed into pre-printed holes on both radius and ulna. **h** 3D printed bones are cast in simulation gel; the dorsal aspect of the model is taped to the side of the mold to remain close to the surface. After cooling, the thin layer of gel on the dorsal side is removed. **i** The free side of the hinge is screwed to the wooden block that serves as the hand (oriented so the wrist can be displaced dorsally) and the model is inserted into the simulation arm. Talcum powder facilitates placement into the skin

To build our distal radius fracture model, we obtained an anatomically correct radius and ulna model from the 3D printing open source site ThingiverseⓇ (www.thingiverse.com, Mountain View, CA, USA) and subsequently uploaded this file into a 3D printing editing website TinkerCad Ⓡ (www.tinkercad.com, San Rafael, CA, USA). Using the editing software, we created a fracture line through the distal radius to model a distal radius fracture. The fracture pieces can be found on TinkerCad under User ID “wdixonPC6LL” as an openly shared file (Fig. 1a). Multiple anchor points through both pieces of the fracture were tested in the model to allow for a dynamic connection between the pieces; these points were optimized for tension and displacement. The bones were printed using an XYZ DaVinci Pro 3D printer (XYZ Printing, San Diego, CA, USA) with ABS filament, though could be printed from any 3D printer with a large enough print bed (Fig. 1b). We found that including a support brim and utilizing the “slow print speed for small parts” setting increased stability of the printed model; additional settings are found in Appendix 1 of the Electronic Supplementary Material. The radius fragments were connected using rubber O‑ring material through the premade dorsal aspect anchor points and secured with zip-ties to create a resistance force to the reduction (Fig. 1c, d).

The bones were then secured on the volar aspect with an inflexible string; we recommend fishing line or a single filament string with good tensile strength to create a point of maximum displacement of the bone fragments (Fig. 1e, f). We secured the radius and ulna at the distal end with a hinge (1.5 inch or 3.81 cm) screwed into pre-made screw holes (Fig. 1g). The attached bones were set in a custom 3D mold (also found on TinkerCad under the same user identification as above) that can be filled with gelatin or medical simulation gel to simulate the soft tissues of the arm (Fig. 1h). We found the medical simulation gel to be more durable, reusable, and clean; this will likely decrease costs over time and allows for longer term storage. After the gel is set, a section of the gel can be peeled off the dorsal aspect so the bone can be palpated under the skin and to allow for fracture articulation. The hinged pieces are subsequently attached to a block of wood (dimensions: 3.5 × 3 × 0.75 inches, or 8.89 × 7.62 × 1.9 cm), which replicates the hand (Fig. 1i). This attachment allows the hand to be placed under traction to assist with a realistic reduction. The entire model is placed into a rubber intravenous catheter training arm (Anatomy Warehouse, Evanston, IL, USA) to obscure details of the fracture model and to simulate a closed reduction performed at the bedside (Fig. 1i). Excluding the cost of the 3D printer, the total cost of the model was approximately USD $140; this includes some items that are used for multiple models, and the most expensive parts are reusable. See Appendix 2 of the Electronic Supplementary Material for a complete list of costs and examples of each piece of the model.

## Evaluation

We examined the fidelity of the distal radius fracture model as part of a simulation-based mastery learning curriculum. The Stanford University Institutional Review Board approved this study. The mastery standard was defined as the minimum passing score for the course, as determined by an expert panel using the Mastery Angoff approach [10]; the minimum passing score was 90% of the total 41 checklist items. We trained all first-year emergency medicine residents (*n* = 15) and first-year orthopedic surgery residents (*n* = 7) at our institution to perform a distal radius fracture reduction using our model and checklist, at the beginning of their residency. First-year trainees are novices and none of our participants had prior formal training in this procedure, which is typical when entering residency. All participants achieved the minimum passing score at the end of our course. Instructors included emergency medicine attendings and orthopedic surgery chief residents.

Course participants and instructors were surveyed regarding the fidelity of the model. The survey tool was created by the author team, reviewed and edited by members of the expert panel who participated in the standard setting exercise, and was piloted in this study. The tool included statements regarding fidelity of the model and confidence in future performance of the procedure, using a Likert-style scale of Strongly Agree to Strongly Disagree [Appendix 3 of the Electronic Supplementary Material]. The majority of participants strongly agreed or agreed that the model had high fidelity (86%, *n* = 22) and that its use in a simulation-based mastery learning course prepared them to treat patients with distal radius fractures (91%, *n* = 22). Each experienced instructor (*n* = 4, excluding author WD) agreed or strongly agreed that the model realistically simulates the reduction of a displaced distal radius fracture and that it is useful in teaching the procedure, although feedback suggested we improve the tactile feel of the model for examination. Responses from instructors were also mixed on the fidelity of the model for the purpose of hematoma blocks, which we plan to address in future models. One advantage of the simulation skin that we used is that it hides needle tracks, requiring students to palpate the model for an accurate hematoma block, instead of following prior insertion points. Our team plans to trial other options for skin and hematoma blocks in second-generation models.

## Reflection

Reduction of a distal radius fracture is a common bedside procedure in the emergency department and there is a clearly demonstrated need to improve competence for this procedure among emergency physicians in the United States. We successfully addressed this problem by developing a novel 3D printed distal radius fracture model to facilitate instruction in reduction techniques using simulation-based mastery learning. Our model is an effective simulator with positive assessments of fidelity for use in training by both residents and faculty members. While 3D printers can be costly to purchase, the separate printing material is not expensive; many universities offer access to 3D printers for teaching purposes, such as the one we described.

The experience of creating a 3D printed distal radius fracture model allowed for professional collaboration and enthusiasm among a diverse group of team members. Our team received positive feedback from emergency medicine and orthopedic surgery residents who applied the knowledge and skills learned in the mastery learning course at the bedside soon thereafter, and residents continue to proudly share accounts of successful fracture reductions.

Challenges encountered in this project centered around early testing and optimization of the model. We will continue to refine our simulator as new options for more flexible skin become commercially available; for example, a negative mold with poured silicone skin [11]. Next-generation versions of our model will include the placement of a catheter through the hollow lumen of the bone to insert moulage blood, to better replicate a hematoma block. Limitations of this project include fidelity testing at a single site and no assessment of patient- level translational outcomes. Next-generation versions of our model and training curriculum will be assessed for patient-level outcomes and we hope to include multiple academic sites.

Our models are now available in an *in situ* simulation room in our emergency department for just-in-time learning and practice prior to performing the procedure at the bedside. Our team continues to develop a suite of new, realistic simulators for teaching other common orthopedic procedures.

## Caption Electronic Supplementary Material

Appendix 1: 3D Printing Settings

Appendix 2: Cost of Model Components

Appendix 3: Post-Survey
