# Supplementary material for: Development of a 3D printed simulator for closed reduction of distal radius fractures
Source: Perspect Med Educ. 2020 Sep 28;10(3):192–5. doi: 10.1007/s40037-020-00609-w (PMC8187689; doi:10.1007/s40037-020-00609-w)
Supplement: Supplementary file 1 — Appendix 1: 3D Printing Settings [file 40037_2020_609_MOESM1_ESM.docx]

**Appendix 1: 3-D Printing Settings**

| Parameter: | Value: |
| --- | --- |
| Layer Height | 0.3 mm |
| Shell Thickness | 4 Layers |
| Fill Density | 10% |
| Nozzle Size | 0.4mm |
| Print Speed | 30mm/s |
| Print Temperature | 210 deg C |
| Print Bed Temperature | 90 deg C |
| Bottom Layer Speed | 20 mm/s |
| Supports | Low Density |
| Material | ABS |
