# Supplementary material for: Development of a 3D printed simulator for closed reduction of distal radius fractures
Source: Perspect Med Educ. 2020 Sep 28;10(3):192–5. doi: 10.1007/s40037-020-00609-w (PMC8187689; doi:10.1007/s40037-020-00609-w)
Supplement: Supplementary file 2 — Appendix 2: Cost of Model Components [file 40037_2020_609_MOESM2_ESM.docx]

**Appendix 2: Cost of Model Components**

| **Item** | **Sample Cost (in US Dollars)** | **Sample Purchase Website** |
| --- | --- | --- |
| 3-D Printer | $300 - 10,000 (one time cost) | <https://www.xyzprinting.com/en-US/home> |
| Plastic Printing Material | $20/spool (each makes multiple models) | <https://www.xyzprinting.com/eshop/en-US/product/ABSFilament1kg> |
| O-ring | $3 | <https://www.lowes.com/pd/Danco-14-Pack-Assorted-in-x-Assorted-in-Rubber-Faucet-O-Ring/1082117> |
| Block of Wood  Dimensions: 3.5x3x.75 inches (8.89x7.62x1.9 cm) | Minimal | Hardware store |
| 1.5 inch (3.81 cm) Hinge with Screws | $3 | <https://www.lowes.com/pd/Gatehouse-1-1-2-in-Zinc-Mortise-Door-Hinge-2-Pack/50041808> |
| Fishing Line | $24 | <https://www.amazon.com/KastKing-SuperPower-Braided-Fishing-Line/dp/B00C6OUE2W?ref_=Oct_BSellerC_3473311_0&pf_rd_p=e9d556a6-0f29-5a96-9937-f9473bbacc85&pf_rd_s=merchandised-search-6&pf_rd_t=101&pf_rd_i=3473311&pf_rd_m=ATVPDKIKX0DER&pf_rd_r=1YGGR1G7THFD23PA5QDS&th=1&psc=1> |
| Zip Ties | $5 | <https://www.lowes.com/pd/Utilitech-100-Pack-4-in-Nylon-Cable-Ties/50005718> |
| Simulation Arm | $60/unit | <https://www.anatomywarehouse.com/replacement-arm-skin-for-deluxe-stat-iv-arm-trainer-simulator-a-103353?gclid=EAIaIQobChMI_aLMnKDp4QIVElcNCh3XLw7xEAQYAiABEgIloPD_BwE> |
| Medical Simulation Gel | $22/pound; 1 pound/model | <https://humimic.com/product/gelatin-2-ballistic-gelatin-by-the-pound/> |
