# Supplementary material for: Development of a 3D printed simulator for closed reduction of distal radius fractures
Source: Perspect Med Educ. 2020 Sep 28;10(3):192–5. doi: 10.1007/s40037-020-00609-w (PMC8187689; doi:10.1007/s40037-020-00609-w)
Supplement: Supplementary file 3 — Appendix 3: Post-Survey [file 40037_2020_609_MOESM3_ESM.docx]

**Appendix 3: Post-Survey**

Simulation-Based Mastery Learning to Teach Distal Radius Fracture Reduction POST-SURVEY

Participant ID:

Two Digit Month of Birth: __ __

Four Digit Random Number (same as the rest of this study) : __ __ __ __

Please choose the appropriate answer choice which best corresponds with how you feel about the statement. Choose only ONE response for each statement.

| 1.In regards to a distal radius fracture, I feel comfortable with: | Strongly Disagree | Disagree | Neutral | Agree | Strongly Agree |
| --- | --- | --- | --- | --- | --- |
| ...examining the hand and wrist for emergent complications of fracture (open, neurovascular injury). | ☐ | ☐ | ☐ | ☐ | ☐ |
| ...reviewing radiography. | ☐ | ☐ | ☐ | ☐ | ☐ |
| ...deciding when it needs further consultation. | ☐ | ☐ | ☐ | ☐ | ☐ |
| ...performing local anesthesia (hematoma block). | ☐ | ☐ | ☐ | ☐ | ☐ |
| ...reducing the fracture. | ☐ | ☐ | ☐ | ☐ | ☐ |
| ...splinting the fracture. | ☐ | ☐ | ☐ | ☐ | ☐ |
| ...reviewing post-reduction x-rays. | ☐ | ☐ | ☐ | ☐ | ☐ |
| ...re-examining post application of splint. | ☐ | ☐ | ☐ | ☐ | ☐ |
| ...delivering discharge instructions and return precautions. | ☐ | ☐ | ☐ | ☐ | ☐ |
| 2. Today’s short, simulation-based class with a hi-fidelity distal radius fracture model increased my confidence in managing the fracture in the ED. | ☐ | ☐ | ☐ | ☐ | ☐ |
| 3. The feedback I received in this training was useful for my learning. | ☐ | ☐ | ☐ | ☐ | ☐ |
| 4. The model realistically simulates a distal radius fracture. | ☐ | ☐ | ☐ | ☐ | ☐ |
| 5. Using the model prepared me to treat patients with distal radius fractures. | ☐ | ☐ | ☐ | ☐ | ☐ |
| 6. Improving my skill and competence in managing distal radius fractures today makes me less likely to request a consult or assistance from a senior when performing this procedure independently. | ☐ | ☐ | ☐ | ☐ | ☐ |
